# Identifying Genetic Variation for Alcohol Dependence

**DOI:** 10.35946/arcr.v34.3.02

**Published:** 2012

**Authors:** Arpana Agrawal, Laura J. Bierut

**Affiliations:** **Arpana Agrawal, Ph.D.,***is an assistant professor in the Department of Psychiatry, Washington University School of Medicine, St. Louis, Missouri.*; **Laura J. Bierut, M.D.,***is a professor in the Department of Psychiatry, Washington University School of Medicine, St. Louis, Missouri.*

**Keywords:** Alcoholism, alcohol dependence, alcohol-metabolizing genes, genetic factors, genetic mapping, genome-wide association studies, candidate gene studies, genetic variants, alcohol dehydrogenase (ADH), aldehyde dehydrogenase (ALDH), alcohol-related cancers, esophageal cancer, γ-aminobutyric acid (GABA), DNA

## Abstract

Researchers are using various strategies to identify the genes that may be associated with alcoholism. The initial efforts primarily relied on candidate gene and linkage studies; more recently, however, modern advances in genotyping have resulted in widespread use of genome-wide association studies for alcohol dependence. The key findings of the earlier studies were that variations (i.e., polymorphisms) in the DNA sequences of the genes encoding alcohol dehydrogenase 1B (i.e., the ADH1B gene), aldehyde dehydrogenase 2 (i.e., the ALDH2 gene), and other alcohol-metabolizing enzymes mediate the risk for alcoholism; moreover, these polymorphisms also have an impact on the risk of alcohol-related cancers, such as esophageal cancer. In addition, a gene encoding one of the receptors for the neurotransmitter γ-aminobutyric acid (GABA) known as GABRA2 seems to have a role in the development of alcohol dependence. Genome-wide association studies now offer a host of emerging opportunities, as well as challenges, for discovering the genetic etiology of alcohol dependence and for unveiling new treatment strategies.

Over the last decade, three large-scale projects have catalyzed a revolution in genetic technologies and studies. First, the Human Genome Project laid the foundation for modern genetic studies of disease by determining the basic sequence of the 3 billion building blocks (i.e., base pairs) that make up the human genome and by identifying the approximately 25,000 genes included in this sequence (www.ornl.gov/sci/techresources/human_genome/home.shtml). A key component of the Human Genome Project was its collaborative nature, with participating researchers sharing their data within 24 hours of generation. A draft sequence of the human genome was released in 2000 with much fanfare, and the project was completed in 2003 with the release of the final sequence data. Second, the International HapMap Project shortly followed behind as a multicountry effort to identify and catalog common genetic similarities and differences across populations (http://hapmap.ncbi.nlm.nih.gov/). This project built on the knowledge that 99.5 percent of the DNA sequence in humans is identical amongst individuals, and that it is only the remaining 0.5 percent that contribute to the development of diseases and differences in traits. The HapMap project mapped these common differences in the genome and, in 2007, published the human haplotype map with over 3 million identified human genetic variants. Finally, since 2008, the 1000 Genomes Project seeks to discover and more finely catalogue genetic variation, particularly those variants that occur at lower frequencies in different populations (www.1000genomes.org). In addition, this project expands the study of human populations around the world to capture more genetic diversity. The information developed from these three projects has transformed the field of genetics and led to genome-wide association studies (GWASs), which aim to identify regions of the genome that are associated with diseases.

GWASs analyze the presence of hundreds of thousands, or even millions, of polymorphisms across a person’s genome. The goal of GWASs is to identify those variants that occur more frequently in people with an illness (e.g., alcoholism) than in those without the disease. Standard GWASs testing now is available, which can query over a million variants that differ in only a single DNA building block (i.e., single nucleotide polymorphisms [SNPs]), indexing variations in the human genome and thereby providing relatively easy access to an individual’s genetic makeup (i.e., genotype). Because the cost of genetic testing has decreased with standardization and mass production, GWASs are designed as large-scale studies to examine genetic variation in thousands to tens of thousands of people. These types of studies already have uncovered thousands of genetic variants that alter the risk of developing many complex diseases, including type 2 diabetes, Crohn’s disease, and Parkinson’s disease (Hindorff et al. 2009). Recently, GWASs also have been applied to the study of alcohol dependence, resulting in the discovery of additional genes that join the existing candidate gene literature for alcohol dependence. This review will place GWASs in the context of the history of the genetic examination of alcohol dependence.

## Role of Alcohol-Metabolizing Genes

Alcohol dependence was one of the first disorders to be associated with a genetic contribution. In 1972, facial flushing and decreased tolerance after alcohol exposure was observed in subjects of Asian ancestry (Wolff 1972)—a response that is associated with characteristic alterations in alcohol metabolism. Upon ingestion and absorption into the blood stream, alcohol first is converted to acetaldehyde in the liver in a process catalyzed by the enzyme alcohol dehydrogenase (ADH). Acetaldehyde is a highly toxic cancer-inducing substance (i.e., carcinogen) that normally is converted rapidly to acetate, a less toxic form. This reaction is mediated by the mitochondrial enzyme aldehyde dehydrogenase (ALDH). In this metabolic chain of events, two basic mechanisms can result in the accumulation of acetaldehyde in the body—faster metabolism of alcohol to acetaldehyde, which is related to increased ADH activity, and/or slower metabolism of acetaldehyde to acetate, which is caused by decreased ALDH activity. The excessive production and accumulation of acetaldehyde then results in the flushing response, which may be accompanied by lightheadedness, nausea, accelerated heart rate, and headaches. Because of the unpleasantness of this reaction, people experiencing flushing typically drink little or no alcohol.

Two key functional polymorphisms, both of which are common in Asian populations, have been implicated in the flushing response to alcohol and consequently identified as protective influences on alcohol consumption and dependence. In the *ADH1B* gene,^1^ a polymorphism called rs1229984 (also referred to as Arg48His), which differs from the normal, or wild-type, DNA sequence in a single nucleotide, results in an amino acid change at position 48 in the β subunit of alcohol dehydrogenase from arginine to histidine (Edenberg 2007). The gene variant (i.e., allele) that encodes histidine in place of arginine at amino acid 48 is called ADH1B^*^2; the resulting enzyme leads to accelerated oxidation of alcohol to acetaldehyde and, consequently, increased acetaldehyde accumulation after alcohol consumption. It has been linked to reduced alcohol consumption and a reduced risk of alcoholism. For example, in a recent meta-analysis of published studies, Li and colleagues (2011) reported that the association between rs1229984 and alcoholism is highly significant (*P* < 10^−30^) in Asian populations. Although the rs1229984 variant is common in populations of Asian descent, it is found infrequently (i.e., in less than 5 percent of individuals) in populations of European or African descent. Bierut and colleagues (2012) recently genotyped and determined the influence of this variant in three large European and African American case–control studies. The results indicated a strong protective effect of rs1229984 for alcohol dependence (*P* = 6.6 × 10^−10^). The allele also was strongly associated with lower alcohol consumption, defined as the maximum number of drinks consumed in a 24-hour period (*P* = 3 × 10^−13^). Thus, although rs1229984 is rare among European and African populations, at an individual level the effect of this *ADH1B* variant on amount of alcohol consumed and on the risk of developing alcohol dependence is the same regardless of ethnicity.

Likewise, a variant called rs671 in the *ALDH2* gene results in an amino acid change from glutamic acid to lysine at position 504 (Glu504Lys) in the ALDH enzyme. The allele containing this variant, called ALDH2^*^2, is associated with substantially reduced ALDH activity. As a result of this inactivation, acetaldehyde accumulates after alcohol consumption and a flushing response occurs, which in turn leads to a reduced likelihood of alcoholism. For instance, Thomasson and colleagues (1991) found that only 12 percent of Chinese men with alcoholism were ALDH2^*^2 carriers (i.e., had one or two copies of the allele), compared with 48 percent of nonalcoholic men. Although this variant is common in Asian populations, it rarely occurs in populations of European and African ancestry.

In addition to the widely replicated associations between the rs1229984 and rs671 with alcoholism risk, certain variants in other genes encoding alcohol-metabolizing enzymes, such as *ADH1C*, *ADH4*, and *ADH5*, also have been linked to alcohol dependence in other populations. Alterations in the *ADH1C* gene, which encodes the γ subunit of alcohol dehydrogenase, can change alcohol metabolism by the encoded protein and have been associated with alcoholism, although these effects are not as pronounced as those noted with ADH1B^*^2 (Edenberg 2007). For instance, the *ADH1C* variants rs698 and rs1693482 also alter the amino acid sequence of the encoded protein and occur with high frequency in European American populations (minor allele ≈ 47 percent). Likewise, variants in *ADH4* and *ADH5* sporadically have been linked to alcoholism. For example, Macgregor and colleagues (2009) reported associations between SNPs in these genes and alcohol dependence symptoms as well as quantity, frequency, and maximum drinks, whereas two other independent studies reported highly significant associations between *ADH4* variants and alcohol dependence (Edenberg et al. 2006; Luo et al. 2006). The study by Mcgregor and colleagues (2009) also is among the few studies of non-Asian populations that demonstrate an association between *ADH* and *ALDH2* polymorphisms and problem drinking.

In summary, genetic variations in many of the genes encoding alcohol-metabolizing enzymes contribute to differences in alcohol intake and, thus, the risk for development of alcohol dependence. The frequency of these genetic variants differs dramatically across human populations of Asian, African, and European ancestry. Some of these genetic effects on alcohol metabolism are strongly correlated with differences in the risk of developing alcohol dependence, whereas others have a more modest effect.

## Candidate Gene Studies

Other early genetic studies of alcohol dependence relied on candidate gene association studies and genome-wide linkage studies. Candidate gene studies leveraged the earliest identified genetic variations in specific genes by examining one gene, and often one variant, at a time to determine whether the variant was associated with alcoholism. These experiments identified hundreds of genes as potentially contributing to alcoholism. For example, in addition to the genes encoding alcohol-metabolizing enzymes, genes involved in brain signaling (i.e., neurotransmitter) systems, such as the dopaminergic, cholinergic, and serotonergic systems, have been nominated for their association with alcohol dependence. In contrast to the alcohol-metabolizing genes, however, these other candidate gene associations have not yet been validated in the modern large-scale genetic studies. This lack of convergence of candidate gene studies and GWASs potentially reflects a large number of false-positive findings from the previous candidate gene findings. Another potential explanation is that extensive genetic heterogeneity exists, meaning that multiple genes each modestly contribute to the development of alcohol dependence and that the samples collected to date, although they were obtained from thousands of subjects, are not yet sufficient in size to detect variation in these regions.

## Genome-Wide Linkage Studies

Genome-wide linkage studies, which examine whether large genetic regions distributed across the entire genome are cotransmitted along with a disease within families, were the first genetic studies to query the entire genome with hundreds or thousands of genetic markers. These studies had two main characteristics: they were family based, and the marker coverage across the genome was modest. Although these linkage studies implicated multiple regions of the genome as being involved in the development of alcoholism, the most consistent findings have pointed to two regions on chromosome 4, with peaks at chromosome locations 4p13–4p12 and on 4q21–4q23.^2^ The 4q21–4q23 region covers the cluster of alcohol dehydrogenase genes that includes, among others, *ADH1B*, supporting the candidate gene findings that alcohol-metabolizing genes are associated with alcohol dependence. The chromosome 4p13 region also contains a cluster of genes encoding the α-subunit of the receptor for the inhibitory neurotransmitter γ-aminobutyric acid (GABA), suggesting that polymorphisms in this receptor also may be involved in the development of alcohol dependence. This cluster of GABA receptor genes is of particular interest because of their putative biological relevance to alcohol-related behaviors, as discussed in the following section.

### Alcohol Use Disorders and GABRA2

Several SNPs in a gene called *GABRA2*, which encodes the α2-subunit of the GABA receptor, have been associated with alcohol dependence. The first study to implicate *GABRA2* in the etiology of alcoholism was conducted by Edenberg and colleagues (2004), who found an association between multiple SNPs (in moderate to high correlation with each other) and alcohol dependence. These results initially were replicated by multiple independent efforts (Drgon et al. 2006; Fehr et al. 2003; Lappalainen et al. 2005; Soyka et al. 2008); however, other investigators could not replicate the findings (e.g., Lind et al. 2008). In contrast to the variants in the alcohol-metabolizing genes, the *GABRA2* variants that are associated with alcohol dependence do not change the amino acid sequence in the encoded protein but instead likely alter the regulation of *GABRA2* protein production.

Because the genetic variants do not change the protein structure of the GABA-α2 subunit, the associations between these genetic variants and alcoholism are less obvious than those for the alcohol-metabolizing genes. Recent laboratory research has focused on the specific functional aspects of *GABRA2* in the etiology of alcoholism. *GABRA2* encodes a subtype of one of five subunits that form the commonly occurring GABA_A_ ionotropic receptor. Binding of GABA to this receptor results, among several outcomes, in sedation and relief of anxiety (i.e., anxiolysis). This anxiolytic effect is more closely related to the α2-subunits than to other subunits (e.g., Dixon et al. 2008). Most α2-containing GABA receptors also are key binding sites for benzodiazepines, whereas receptors containing other subunits only show sensitivity to ethanol. The genetic variants in the *GABRA2* gene do not result in functional changes in the receptor, and, in fact, little is known about how the variants that are related to the development of alcohol dependence influence *GABRA2* activity. Some investigators (Haughey et al. 2008) demonstrated that a variant called rs279858 resulted in changes in the levels of an intermediary molecule formed during the conversion of the genetic information encoded in the gene into a protein product (i.e., in mRNA levels). Kareken and colleagues (2010) found variations in the brain’s response^3^ to preferred alcoholic drink aromas in a study comparing people who carried two copies (i.e., were homozygotes) or only one copy (i.e., were heterozygotes) of the “risk” allele for a variant called rs279871. Finally, Villafuerte and colleagues (2012) reported that two *GABRA2* variants called rs279826 and rs279858, which previously had been implicated in the etiology of alcoholism, not only correlated with impulsiveness but also with activity in a brain region called the insula cortex during monetary reward and loss. Thus, in experiments assessing responses during reward anticipation and loss, study participants from alcoholic families with the haplotype that is associated with increased alcoholism vulnerability showed significantly higher activation in the left insula than did participants with a different haplotype. However, much more research is needed to understand the biology underlying these genetic associations with *GABRA2.*

Animal models also have begun to address these functional aspects of *GABRA2*, as follows:

- Work with frog eggs (i.e., *Xenopus* oocytes) has demonstrated that the α2 subunits are involved in regulating GABA currents upon ethanol exposure (Hurley et al. 2009).
- Studies of mice that had been genetically modified to produce or not produce the GABA-α2 subunit (i.e., knock-in and knock-out mice) demonstrated the important role of this subunit in anxiolysis (Boehm et al. 2004; Dixon et al. 2008). Additional studies with these animals provided evidence for the involvement of the α2 subunits in the hypnotic effects of ethanol when administered together with the benzodiazepine diazepam (Tauber et al. 2003).
- In analyses comparing alcohol-preferring (P) and non-preferring (NP) rats (McBride and Li 1998), Liu and colleagues (2011) reported that P rats show elevated levels of GABA-A subunits, including the α2 subunits, in a brain region called the central nucleus of the amygdala. Intriguingly, when molecules that specifically could inhibit the *GABRA2* gene (i.e., α2 silent RNA [siRNA]) were introduced into the central amygdala, even the P animals no longer demonstrated binge drinking for a short period of time, and this effect correlated with a significant reduction of α2 levels. This effect did not occur when siRNA for another receptor subunit (i.e., α1 siRNAs) was used or when α2 siRNA was introduced in other control regions of the brain.

Thus, much research has examined the role of the α-subunit of the GABA receptor, and this protein clearly plays a central role in alcohol-mediated anxiolysis and other effects of alcohol use. Nevertheless, it remains important to more clearly connect genetic variations found to be associated with alcoholism risk in humans with basic biologic function. Functional studies using animal models, postmortem brain tissue, and novel methodologies that examine gene expression in the context of neuronal connectivity in alcoholics and nonalcoholics are among a few methods that may serve to further elucidate the function of candidate variants identified in human gene-association studies.

## GWASs

GWASs represent the most recent paradigm change for gene discovery. The allure of GWASs is that they allow for interrogation of hundreds of thousands to over 1 million SNPs across the human genome at relatively modest cost. Thus, GWASs can potentially lead to the identification of variants of modest effect size that may not be recognized in candidate gene studies for their biological significance in alcoholism. The principal disadvantage is the fairly severe multiple-testing burden (i.e., the need to account for the possibility that when 1 million tests are conducted, some positive results will be obtained due to chance alone) imposed by GWASs, which results in the requirement for statistical significance to be denoted by *P* values of 5 × 10^−8^ and lower. This represents a very high significance level for validation; consequently, many SNPs that truly are associated with alcoholism risk may not yet have been recognized because they have not yet surpassed this threshold.

Four GWASs of alcohol dependence and two of alcohol consumption now have been completed in populations of European ancestry. In the first of these, Treutlein and colleagues (2009) found several SNPs with *P* values in the range of 10^−6^; upon pooling with a replication sample, two of these SNPs, rs7590720 and rs1344694, showed statistical significance in the combined male-only sample of 1,460 alcohol-dependent subject and 2,332 community control subjects. These SNPs are located in a region of the long arm of chromosome 2 where prior linkage studies have identified increased allele-sharing for alcoholism. The gene closest to the association signal, *PECR* (which encodes the enzyme peroxisomal trans-2-enoyl-CoA reductase) is involved in the metabolism of fatty acids, particularly during deprivation when energy expenditure transitions from carbohydrates to fatty acids. In addition, the investigators noted that a SNP called rs1614972 in the *ADH1C* gene was strongly associated with alcohol dependence (*P* = 1.4 × 10^−4^) even though it did not meet strict standards for genome-wide multiple testing. Two subsequent studies have identified a SNP in *PKNOX2* (rs10893366, *P* = 1.9 × 10^–7^) and a cluster of SNPs on chromosome 11 (*P* < 10^−5^) that also are associated with alcohol dependence but did not meet the level of statistical significance (Bierut et al. 2010; Edenberg et al. 2010). A fourth GWASs of quantitative indices of excessive alcohol consumption recently was completed in a large cohort of Australian families, in which no genetic variant met the standards for genome-wide significance (Heath et al. 2011). Across the four studies, there was no evidence for replication of any genetic signals, raising the possibility that the identified signals were false positives or that true signals could not be detected as a result of the (by GWASs standards) relatively small sample sizes (i.e., several thousand subjects).

Although GWASs of subjects of European descent have struggled to identify and replicate SNPs associated with alcohol dependence, a recent GWASs of a sample of 1,721 Korean men reported associations with *P* values of 10^−58^ and lower for SNPs in moderate to high linkage disequilibrium^4^ with SNPs in the *ALDH2* gene, including rs671 (Baik et al. 2011). In this study, carriers of the protective allele of the SNP rs11066280 in the gene *C12orf51*, which frequently occurs together with rs671 in *ALDH2* (the SNP that inactivates *ALDH2*), had a 13-fold increased risk for alcohol flushing and a corresponding 5-fold decreased likelihood of developing alcohol problems consistent with alcohol dependence. Because this variant is rare in subjects of European and African descent, this strong finding is not seen, nor expected, in non-Asian populations.

In addition to studying alcohol dependence, a strategy to increase the power of GWASs has been to examine alcohol consumption as a quantitative variable. A recent meta-analysis of alcohol consumption by Schumann and colleagues (2011) used results from an initial sample of 26,316 population-based subjects and a follow-up sample of 21,185 European American subjects to identify a SNP called rs6943555 in the autism susceptibility candidate 2 (*AUTS2*) gene that was associated with alcohol consumption (in grams/day/kilogram of body weight) at *P* < 5 × 10^−9^. Specifically, people who carried the minor allele of rs6943555 showed 5.5 percent lower alcohol consumption than people who carried the major allele; however, secondary analyses did not reveal any association with alcohol dependence. Follow-up studies showed changes in mRNA expression that correlated with the rs6943555 genotype; moreover, the gene itself was expressed differently in low-alcohol– versus high-alcohol–preferring rats. Likewise, Heath and colleagues (2011) examined a quantitative factor score created from items assessing quantity and frequency of consumption, frequency of intoxication, and maximum drinks in a single 24-hour period, all reported for the 12-month period when the participants indicated that they drank the heaviest. The results implicated variants in the *TMEM108* gene, which encodes a transmembrane protein of unknown function (*P* = 1.2 × 10^−7^). These two initial studies of alcohol consumption implicate different genomic regions, and their findings have not converged. With larger sample sizes, it will become clearer whether these results represent true findings.

## Genetic Studies of Alcoholism in the Context of Other Diseases

Alcohol is consumed by an overwhelming majority of the U.S. population, and 55 percent of the world’s population has consumed alcohol (World Health Organization [WHO] 2011). Per capita consumption varies greatly by country. For example, the most recent figures from the WHO (2011) indicate that among people ages 15 and older, per capita annual alcohol consumption was 11 liters in Russia and 8.5 liters in the United States, with much lower consumption levels reported in parts of Asia and northern Africa. Although modest alcohol consumption is associated with health (e.g., cardiovascular) benefits, according to the WHO, 2.5 million people die each year from the harmful effects of alcohol (http://www.who.int/substance_abuse/facts/en/). In the United States, nearly 80,000 people die annually from the short- and long-term consequences of alcohol use (Mokdad et al. 2004). Excessive alcohol consumption can lead to alcohol dependence, which affects 12.5 percent of people in the United States across their lifetime (Hasin et al. 2007). Thus, the health effects of alcohol consumption remain a public health priority that needs to be studied from all angles, including improving prevention and treatment, while also examining basic biological underpinnings.

Alcohol consumption increases the likelihood of several other disorders. For example, population-based studies have demonstrated that esophageal cancer is linked to alcohol consumption as a result of the tissue’s exposure to ethanol and its metabolite acetaldehyde. Consequently, functional variants in *ADH1B* and *ALDH2* that affect alcohol clearance and acetaldehyde accumulation also play a role in the development of esophageal cancer. The effects of these variants on cancer are strongly moderated by accompanying alcohol exposure. For example, people who carry the ADH1B^*^2 allele that results in more rapid ethanol conversion to acetaldehyde, experience flushing and, hence, have reduced likelihood of alcohol intake, protecting them from esophageal cancer. Conversely, people who carry the ADH1B^*^1 variant, which results in slower alcohol metabolism, are at increased risk for esophageal cancer, arguably because of extended tissue exposure to ethanol. Likewise, having the ALDH2^*^2 allele, which inactivates aldehyde dehydrogenase, has protective effects on both alcohol intake and esophageal cancer. However, people who carry only one copy of ALDH2^*^2 often continue to drink, which leads to acetaldehyde accumulation and consequently increases the risk for esophageal cancer (Brooks et al. 2009).

Because both protective gene variants are more common in Asian populations, several studies have demonstrated the influence of ADH1B^*^2 and ALDH2^*^2 on esophageal cancer in these populations (Ding et al. 2010; Hashibe et al. 2008). A meta-analysis of published studies on Asian populations (Yang et al. 2010) further confirmed that the potentially large effect of these gene variants on esophageal cancer was exacerbated by alcohol exposure.

A recent GWASs of cancers of the upper aerodigestive tract (UADT) (i.e., cancers of the oral cavity, pharynx, larynx, and esophagus) identified SNP rs1229984 in *ADH1B* as a risk variant for esophageal cancer in samples with European ancestry (McKay et al. 2011). This variant is present in as many as 70 to 80 percent of people in Asian populations (Han et al. 2007; Kosoy et al. 2009) but is found in less than 5 percent of European populations. Nevertheless, it is by far the strongest genetic variant that alters the risk for the development of UADT cancer, even after accounting for alcohol consumption. Variants in other alcohol-metabolizing genes also have been implicated in the development of esophageal cancer.

In several of these studies, the effects of *ADH1B* and *ALDH2* variants predominantly were noted in those who drink alcohol. For instance, even in people with two copies of the ALDH2^*^2 allele, which is highly protective for alcohol intake, the few drinkers are at a profoundly elevated risk for esophageal cancer (Yang et al. 2010). It might be hypothesized that this finding reflects a genotype–environment correlation where *ALDH2* and *ADH1B* variants influence the likelihood of continued alcohol (and acetaldehyde) exposure, which, in turn, serves as the environmental risk factor for the development of esophageal cancer, either as a result of prolonged ethanol exposure or of the carcinogenic effects of acetaldehyde. An alternative explanation is that of a genotype × environment interaction—that is, people who carry the *ALDH2* and *ADH1B* genotypes that protect against alcohol intake might be exquisitely sensitive to the joint effects of genotype and alcohol consumption on their vulnerability to esophageal cancer.

## Genes to Function to Treatment

Genetic association studies only are a first step in understanding the biology underlying alcohol dependence. An association represents a correlation with a tested variant (and all the untested correlated variants). These groups of associated SNPs often span many genes on a chromosome, and once a genetic association is confirmed the task is to investigate how these variants and the genes they are located in contribute to the biological mechanisms underlying disease development. The ultimate goal of understanding these biological mechanisms is to gain new insights into potential treatment approaches.

To some extent, clinicians already are exploiting the understanding of genetic variation in the development of alcoholism in their treatment strategies. For example, the variation in enzymatic pathways of alcohol metabolism found in the population is mimicked in the pharmacologic treatment of alcohol dependence using the medication disulfiram, which inhibits ALDH and is a pharmacologic treatment similar to the natural variation related to rs671 and decreased ALDH activity in Asians. If alcohol is consumed after taking disulfiram, acetaldehyde levels increase, causing flushing, nausea, vomiting, and headache in the patient, which produces an aversive response to continued alcohol use.

## Future Directions

Researchers long have known that alcohol dependence clusters in families. During the past 50 years, adoption and twin studies definitively have demonstrated that this clustering is related to genetic influences. Twin studies have estimated that 30 to 60 percent of the variance in alcohol dependence is attributable to the effects of genetic variants segregating in families (Dick and Bierut 2006). Researchers now are entering a new phase in genetic studies where they are beginning to unlock the genetic code that leads to disease.

***“Ebrii gignunt ebrios” (One drunkard begets another)***

***—attributed by Richard Burton to Plutarch***

Although the GWASs approach has been successful for many illnesses, much of the genetic variation underlying the development of alcohol dependence remains undiscovered. Thus, although the heritability of alcohol dependence approaches 50 percent, the explained genetic variance to date is less than 1 percent, which raises the question “Where is the missing genetic variance?” Three main explanations have been given for the missing variance in the genetic risk for alcohol dependence: (1) many genes of small effect may be involved that do not yet surpass the stringent threshold for genome-wide significance; (2) rare variations—that is, SNPs that only occur in less than 1 percent of the population—exist that are not included on standard chips and hence not captured by GWASs genotyping; and (3) other mechanisms, including the interplay of genes and environments, may contribute that are not detected in current analyses.

A key component for the future success of genetic studies is the creation of large-scale genetic research consortia that permit the study of large numbers of comprehensively assessed subjects. Collaboration is necessary for these new genetic studies, and resources are shared with the scientific community through the Database of Genotypes and Phenotypes (dbGaP), which allows investigators to test new hypotheses about the genetic contributions to alcohol dependence (www.ncbi.nlm.nih.gov/sites/entrez?db=gap). This collaborative research is necessary because of the large sample sizes—tens of thousands or more—needed to study genetic effects. The sharing of genetic information has been a corner stone of the Human Genome Project, the International HapMap Project, and the 1000 Genomes Project, and the collaborative aspect must continue if researchers are to gain a better understanding of disease.

The future of genetic studies potentially may help people understand their personal risks and potential treatments for disease. The strongest genetic contributors to alcohol dependence to date are related to the pharmacologic responses to alcohol represented by variations in alcohol-metabolizing genes. This knowledge represents the first level of genetic understanding, and researchers now are poised to move to the next level with the identification of additional genes that contribute to alcoholism. Several challenges remain, however, as this field moves forward. First, investigators must increase the diversity of the populations under study. Varying allele frequencies across populations mean that important genetic contributors in one population may not be seen in another, with the classic example being the variation in the alcohol-metabolizing genes that contributes to alcohol dependence. Increasing diversity in the populations under study will allow researchers to leverage these differences to refine association signals and also to increase the potential discovery of genetic variants. A second challenge is to integrate the findings from candidate gene studies and GWASs. The results from these two methods only have converged modestly, primarily with the alcohol-metabolizing genes, and the discrepancies should be resolved so that the high false-negative rate of GWASs can be balanced with the high false-positive results in candidate gene studies. Finally, the environment will always play an important role in the development of alcoholism by providing the exposure to and availability of alcohol. Therefore, it will be essential to integrate genetic predispositions with environmental exposures in order to better prevent and treat alcohol dependence, one of the Nation’s most devastating illnesses.

## Footnotes

4 The term linkage disequilibrium means that some combinations of alleles or genetic markers in a population occur more often or less often than would be expected from a random formation of haplotypes based on the alleles’ frequencies.

## References

[b1-arcr-34-3-274] Baik I, Cho NH, Kim SH (2011). Genome-wide association studies identify genetic loci related to alcohol consumption in Korean men. American Journal of Clinical Nutrition.

[b2-arcr-34-3-274] Bierut LJ, Agrawal A, Bucholz KK (2010). A genome-wide association study of alcohol dependence. Proceedings of the National Academy of Sciences of the United States of America.

[b3-arcr-34-3-274] Bierut LJ, Goate AM, Breslau N (2012). *ADH1B* is associated with alcohol dependence and alcohol consumption in populations of European and African ancestry. Molecular Psychiatry.

[b4-arcr-34-3-274] Boehm SL, Ponomarev I, Jennings AW (2004). gamma-Aminobutyric acid A receptor subunit mutant mice: New perspectives on alcohol actions. Biochemical Pharmacology.

[b5-arcr-34-3-274] Brooks PJ, Enoch MA, Goldman D (2009). The alcohol flushing response: An unrecognized risk factor for esophageal cancer from alcohol consumption. PLoS Medicine.

[b6-arcr-34-3-274] Dick DM, Bierut LJ (2006). The genetics of alcohol dependence. Current Psychiatry Reports.

[b7-arcr-34-3-274] Ding JH, Li SP, Cao HX (2010). Alcohol dehydrogenase-2 and aldehyde dehydrogenase-2 genotypes, alcohol drinking and the risk for esophageal cancer in a Chinese population. Journal of Human Genetics.

[b8-arcr-34-3-274] Dixon CI, Rosahl TW, Stephens DN (2008). Targeted deletion of the GABRA2 gene encoding alpha2-subunits of GABA(A) receptors facilitates performance of a conditioned emotional response, and abolishes anxiolytic effects of benzodiazepines and barbiturates. Pharmacology, Biochemistry, and Behavior.

[b9-arcr-34-3-274] Drgon T, D’Addario C, Uhl GR (2006). Linkage disequilibrium, haplotype and association studies of a chromosome 4 GABA receptor gene cluster: Candidate gene variants for addictions. American Journal of Medical Genetics. Part B, Neuropsychiatric Genetics.

[b10-arcr-34-3-274] Edenberg HJ (2007). The genetics of alcohol metabolism: Role of alcohol dehydrogenase and aldehyde dehydrogenase variants. Alcohol Research & Health.

[b11-arcr-34-3-274] Edenberg HJ, Dick DM, Xuei X (2004). Variations in GABRA2, encoding the alpha 2 subunit of the GABA(A) receptor, are associated with alcohol dependence and with brain oscillations. American Journal of Human Genetics.

[b12-arcr-34-3-274] Edenberg HJ, Koller DL, Xuei X (2010). Genome-wide association study of alcohol dependence implicates a region on chromosome 11. Alcoholism: Clinical and Experimental Research.

[b13-arcr-34-3-274] Edenberg HJ, Xuei X, Chen HJ (2006). Association of alcohol dehydrogenase genes with alcohol dependence: A comprehensive analysis. Human Molecular Genetics.

[b14-arcr-34-3-274] Fehr C, Rademacher BL, Buck KJ (2003). Evaluation of the glutamate decarboxylase genes Gad1 and Gad2 as candidate genes for acute ethanol withdrawal severity in mice. Progress in Neuropsychopharmacology & Biological Psychiatry.

[b15-arcr-34-3-274] Han Y, Gu S, Oota H (2007). Evidence of positive selection on a class I ADH locus. American Journal of Human Genetics.

[b16-arcr-34-3-274] Hashibe M, McKay JD, Curado MP (2008). Multiple ADH genes are associated with upper aerodigestive cancers. Nature Genetics.

[b17-arcr-34-3-274] Hasin DS, Stinson FS, Ogburn E (2007). Prevalence, correlates, disability, and comorbidity of DSM-IV alcohol abuse and dependence in the United States: Results from the National Epidemiologic Survey on Alcohol and Related Conditions. Archives of General Psychiatry.

[b18-arcr-34-3-274] Haughey HM, Ray LA, Finan P (2008). Human gamma-aminobutyric acid A receptor alpha2 gene moderates the acute effects of alcohol and brain mRNA expression. Genes, Brain, and Behavior.

[b19-arcr-34-3-274] Heath AC, Whitfield JB, Martin NG (2011). A quantitative-trait genome-wide association study of alcoholism risk in the community: Findings and implications. Biological Psychiatry.

[b20-arcr-34-3-274] Hindorff LA, Sethupathy P, Junkins HA (2009). Potential etiologic and functional implications of genome-wide association loci for human diseases and traits. Proceedings of the National Academy of Sciences of the United States of America.

[b21-arcr-34-3-274] Hurley JH, Ballard CJ, Edenberg HJ (2009). Altering the relative abundance of GABA A receptor subunits changes GABA- and ethanol-responses in Xenopus oocytes. Alcoholism: Clinical and Experimental Research.

[b22-arcr-34-3-274] Kareken DA, Liang T, Wetherill L (2010). A polymorphism in GABRA2 is associated with the medial frontal response to alcohol cues in an fMRI study. Alcoholism: Clinical and Experimental Research.

[b23-arcr-34-3-274] Kosoy R, Nassir R, Tian C (2009). Ancestry informative marker sets for determining continental origin and admixture proportions in common populations in America. Human Mutation.

[b24-arcr-34-3-274] Lappalainen J, Krupitsky E, Remizov M (2005). Association between alcoholism and gamma-amino butyric acid alpha2 receptor subtype in a Russian population. Alcoholism: Clinical and Experimental Research.

[b25-arcr-34-3-274] Li D, Zhao H, Gelernter J (2011). Strong association of the alcohol dehydrogenase 1B gene (ADH1B) with alcohol dependence and alcohol-induced medical diseases. Biological Psychiatry.

[b26-arcr-34-3-274] Lind PA, Macgregor S, Agrawal A (2008). The role of GABRA2 in alcohol dependence, smoking, and illicit drug use in an Australian population sample. Alcoholism: Clinical and Experimental Research.

[b27-arcr-34-3-274] Liu J, Yang AR, Kelly T (2011). Binge alcohol drinking is associated with GABAA alpha2-regulated Toll-like receptor 4 (TLR4) expression in the central amygdala. Proceedings of the National Academy of Sciences of the United States of America.

[b28-arcr-34-3-274] Luo X, Kranzler HR, Zuo L (2006). ADH4 gene variation is associated with alcohol dependence and drug dependence in European Americans: Results from HWD tests and case-control association studies. Neuropsychopharmacology.

[b29-arcr-34-3-274] Macgregor S, Lind PA, Bucholz KK (2009). Associations of ADH and ALDH2 gene variation with self report alcohol reactions, consumption and dependence: An integrated analysis. Human Molecular Genetics.

[b30-arcr-34-3-274] McBride WJ, Li TK (1998). Animal models of alcoholism: Neurobiology of high alcohol-drinking behavior in rodents. Critical Reviews in Neurobiology.

[b31-arcr-34-3-274] McKay JD, Truong T, Gaborieau V (2011). A genome-wide association study of upper aerodigestive tract cancers conducted within the INHANCE Consortium. PLoS Genetics.

[b32-arcr-34-3-274] Mokdad AH, Marks JS, Stroup DF, Gerberding JL (2004). Actual causes of death in the United States, 2000. JAMA: Journal of the American Medical Association.

[b33-arcr-34-3-274] Schumann G, Coin LJ, Lourdusamy A (2011). Genome-wide association and genetic functional studies identify autism susceptibility candidate 2 gene (AUTS2) in the regulation of alcohol consumption. Proceedings of the National Academy of Sciences of the United States of America.

[b34-arcr-34-3-274] Soyka M, Preuss UW, Hesselbrock V (2008). GABA-A2 receptor subunit gene (GABRA2) polymorphisms and risk for alcohol dependence. Journal of Psychiatric Research.

[b35-arcr-34-3-274] Tauber M, Calame-Droz E, Prut L (2003). alpha2-gamma-Aminobutyric acid (GABA)A receptors are the molecular substrates mediating precipitation of narcosis but not of sedation by the combined use of diazepam and alcohol in vivo. European Journal of Neuroscience.

[b36-arcr-34-3-274] Thomasson HR, Edenberg HJ, Crabb DW (1991). Alcohol and aldehyde dehydrogenase genotypes and alcoholism in Chinese men. American Journal of Human Genetics.

[b37-arcr-34-3-274] Treutlein J, Cichon S, Ridinger M (2009). Genome-wide association study of alcohol dependence. Archives of General Psychiatry.

[b38-arcr-34-3-274] Villafuerte S, Heitzeg MM, Foley S (2012). Impulsiveness and insula activation during reward anticipation are associated with genetic variants in GABRA2 in a family sample enriched for alcoholism. Molecular Psychiatry.

[b39-arcr-34-3-274] Wolff PH (1972). Ethnic differences in alcohol sensitivity. Science.

[b40-arcr-34-3-274] World Health Organization (2011). Global Status Report on Alcohol and Health.

[b41-arcr-34-3-274] Yang SJ, Yokoyama A, Yokoyama T (2010). Relationship between genetic polymorphisms of ALDH2 and ADH1B and esophageal cancer risk: A meta-analysis. World Journal of Gastroenterology.

